# SPIKES-D: a proposal to adapt the SPIKES protocol to deliver the diagnosis of dementia

**DOI:** 10.1590/1980-57642020dn14-040001

**Published:** 2020-12

**Authors:** Vanessa Giffoni de Medeiros Nunes Pinheiro Peixoto, Rosiane Viana Zuza Diniz, Clécio de Oliveira Godeiro

**Affiliations:** 1 Clinical Medicine Department, Universidade Federal do Rio Grande do Norte – Natal, RN, Brazil.; 2 Internal Medicine Department, Universidade Federal do Rio Grande do Norte – Natal, RN, Brazil.

**Keywords:** Alzheimer disease, dementia, disclosure, diagnosis, communication, doença de Alzheimer, demência, revelação, diagnóstico, comunicação

## Abstract

Dementia is a life-threatening and stigmatizing condition, with devastating impacts on the patient's personal identity and caregivers. There are many barriers to an effective diagnosis disclosure of dementia, including fear of causing distress, uncertainty of diagnosis, caregivers’ objection and lack of training in communication skills in undergraduate medical schools. Although some studies have been published on how to help physicians deliver an Alzheimer's disease diagnosis, no specific protocol has been published yet. The SPIKES protocol is a didactic approach designed to deliver bad news related to cancer, but it has been used globally and in a variety of clinical settings, including the teaching of communication skills to medical students and residents. It is known, however, that the cognitive impairment of Alzheimer's disease and other dementias may limit the understanding of the diagnosis’ complexity; hence, a few adaptations of this model were made after reviewing the current literature on dementia diagnosis disclosure. The suggested SPIKES-D protocol seems to encompass current guidelines about the communication of the diagnosis of dementia, keeping its didactic approach on breaking bad news and helping fulfill the gaps in this topic.

## RESUMO.

A demência é uma condição incurável e estigmatizada, com impacto devastador na identidade pessoal do paciente e seus cuidadores. Existem muitas barreiras para uma adequada comunicação do diagnóstico às pessoas com demência, incluindo medo de causar estresse psicológico, incerteza do diagnóstico, objeção dos cuidadores e falta de treinamento em habilidades de comunicação nas escolas de medicina. Embora alguns artigos sobre como auxiliar a equipe médica a comunicar um diagnóstico de demência tenham sido publicados, nenhum protocolo específico foi publicado até o presente momento. O protocolo SPIKES é uma abordagem didática desenvolvida para auxiliar a comunicação de más notícias relacionadas ao câncer, mas tem sido utilizado em todo o mundo e nos mais diversos contextos clínicos, inclusive no ensino de habilidades de comunicação para estudantes e residentes de medicina. Entretanto, é sabido que o declínio cognitivo inerente à doença de Alzheimer e outras demências pode limitar a compreensão da complexidade do diagnóstico, tendo sido realizadas, portanto, algumas adaptações nesse protocolo após revisão da literatura acerca da comunicação do diagnóstico das demências. O protocolo SPIKES-D aqui sugerido parece englobar as diretrizes atuais sobre a comunicação do diagnóstico de demências, preservando seu caráter didático na comunicação de más notícias e auxiliando no preenchimento das lacunas neste tópico.

## INTRODUCTION

Population aging is among the most important global transformations. In 2017, there were an estimated 962 million people aged 60 or over in the world, comprising 13 per cent of the global population.^1^ Compared to European and North American countries, Latin America (LA) is experiencing this demographic change at a significantly faster rate. Although systematic reviews of studies on the prevalence of dementia have revealed a slightly downward trend for the USA and Europe,^2^ the number of people with dementia in LA is expected to rise from 7.8 million in 2013 to over 27 million by 2050.^3^ Nowadays, the global prevalence of dementia in LA has reached 7.1%, with Alzheimer's disease (AD) being the most frequent type.

In light of these challenging statistics, an increasing number of older people, caregivers and physicians will be involved in the process of diagnosis of AD and other forms of dementia. This life-threatening disease causes devastating impacts on patient's personal identity and their caregivers, besides personal, familial and societal costs. The disclosure of AD poses a great challenge because it involves emotionally charged communication of a stigmatized condition, which is currently one of the most feared diseases.^4^ ^,^ ^5^ In the last decade, a Brazilian survey revealed that less than 50% of geriatricians, neurologists and psychiatrists would regularly inform patients of the diagnosis of AD, in agreement with other studies.^4^ ^–^ ^6^

There are many barriers to an effective disclosure of diagnosis of dementia to patients, including fear of withdrawing hope and eliciting distress and other negative reactions, uncertainty of diagnosis, caregivers’ objection and lack of training on communication skills in medical schools. Physicians also argue that the patients’ cognitive decline may limit the understanding of the diagnosis and finally that there is the patient's right “not to know”.^7^ ^–^ ^9^ However, family caregivers relate positive consequences of knowing the diagnosis, as relief for understanding the origin of the cognitive complaints, improved quality of life and relationship with the patient and opportunity to prepare for the future. The majority of studies found that patients and family caregivers wanted to be informed of the diagnosis of dementia, as well as healthy elderly persons’ preferences concerning this hypothetical diagnosis in the future.^5^ ^,^ ^10^ ^–^ ^14^

In the last two decades, many studies have been published assessing the topic of disclosure of dementia. Initial papers started a debate among clinicians and researchers about the topic of “truth telling” and its benefits to dementia patients, along with investigations of attitudes and preferences of those involved in the process of disclosure.^4^ ^,^ ^7^ ^,^ ^15^ ^–^ ^17^ Later, studies focused more on how this communication is delivered.^5^ ^,^ ^6^ ^,^ ^18^ ^,^ ^19^ Although a few studies have been conducted on guidelines on how to help physicians deliver an AD diagnosis, no specific protocol has been published yet.^20^ ^–^ ^24^ A new tool is still under development and evaluation, and it should meet the clinicians’, patients’ and caregivers’ perspectives in the future.^25^ To date, there are well-established protocols related to bad news communication in other health care settings, especially in the oncology field, which may have transferable concepts to the dementia patient.^26^ ^,^ ^27^

The SPIKES protocol is a didactic approach to deliver bad news related to cancer but it has been used globally and in a variety of clinical settings, including in teaching of communication skills to medical students and residents.^26^ ^,^ ^28^ ^–^ ^30^ It is carried out in a six-stage process, in which the first three stages focus on getting started and identifying how much the patient knows and what they do and do not want to know, highlighting the patient's right not to know. The remaining three stages focus on how the professional shares the diagnosis and are concerned with responding to the individual's feelings and questions, and planning for the future as well. Some authors have previously suggested that it could possibly be adapted for the specific disclosure of dementia, which was the starting point for this work.^5^

It is known, however, that the cognitive impairment of AD and other dementias may limit the understanding of the diagnosis’ complexity; hence, a few adaptations to this model were made after reviewing the current literature on dementia diagnosis disclosure.

## METHODS

To ensure we had a comprehensive view of the delivery of the diagnosis of dementia, we conducted a literature review on PubMed, SciELO and Google Scholar databases between June 2018 and October 2019, using the search terms “bad news”, “dementia”, “diagnosis”, “disclosure”, “deliver”, “communication”, “Alzheimer”. We also searched using the terms “disclos*”, “communicat*” and “deliver*”. We selected papers in English, Portuguese, French and Spanish, especially those that addressed guidelines or suggestions on the approach of an appropriate disclosure of dementia. After obtaining one of the SPIKES authors consent, specialists proposed adaptations of each of the six steps of the SPIKES protocol to adjust it to the dementia care setting. We named the adapted protocol SPIKES-D (SPIKES-Dementia).

## RESULTS

The literature search mainly found papers reporting surveys on clinicians’ attitudes towards the communication of a dementia diagnosis^3^ ^,^ ^6^ ^,^ ^7^ ^,^ ^9^ ^,^ ^16^ ^,^ ^18^ ^,^ ^19^ or patients’ and caregivers’ experiences about receiving a diagnosis,^7^ ^,^ ^10^ ^–^ ^15^ ^,^ ^17^ including two systematic reviews.^4^ ^,^ ^5^ Seven articles were selected considering their focus on practical guidance of reasonable approaches to communicate a diagnosis of dementia.^7^ ^,^ ^8^ ^,^ ^20^ ^–^ ^23^ ^,^ ^25^ Their instructions were meshed with the original SPIKES protocol to adapt it to a dementia setting. One of these articles was an original study and developed a binary protocol by using thematic analysis from interviews with patients, caregivers and clinicians, but it still lacked research to determine its validity.^25^ The other papers suggested flexible guidelines on the basis of literature review of the topic and experts’ opinions. None of them addressed a specific dementia subtype or explored the applicability or success of the guidelines in terms of patient outcome.

### Proposed changes in the spikes protocol (Appendix 1)

Step 1 — setting up the interview

- It is advised to have a trusted family member on the disclosure meeting. Cognitive impairment may limit the true understanding of the diagnosis and its implications. There is an increased importance of a companion in the context of the dementia-care setting, often taking on important roles as informant and patient supporter.^8^ ^,^ ^21^ ^,^ ^22^ ^,^ ^25^
- Avoid social talk or long introductions. The patient needs to focus on the reason of the meeting. These attitudes may confuse the person with dementia, and it may be difficult for them to concentrate on the actual content of the meeting.^21^
- It is advisable to deliver the diagnosis in a multidisciplinary approach. Besides the physician, nurses, social workers or psychologists may be present at this moment.^21^ ^,^ ^22^

Step 2 — assessing the patient's perception

- It is important to identify the extent of cognitive impairment and degree of insight of the patient. An effective communication still occurs in the early stages of dementia or MCI (mild cognitive impairment). As dementia progresses, the patient loses decision-making capacity and has limited ability to understand the diagnosis and its implications.^7^ ^,^ ^8^ ^,^ ^20^ ^–^ ^23^
- It is important to address the person with dementia directly in the disclosure meeting. A common mistake is to communicate mainly with family members or caregivers, neglecting the patient with dementia.^8^ ^,^ ^21^

Step 3 — obtaining the patient's invitation

- Before inviting the patient to know his/her diagnosis, we should inform him/her that after having considered the cognitive complaints, cognitive assessment and laboratory results, there is a probable hypothesis for his problem. An appropriate way to invite him to know the diagnosis should be: “Mr./Mrs.____, we have analyzed the cognitive tests and the laboratory results you have undergone. It seems we already have an idea of what is happening to your memory. Would you like to know it”?^7^ ^,^ ^8^ ^,^ ^20^ ^,^ ^21^
- If a patient declines being told the diagnosis, the clinician should discuss a future talk with a family member.

Step 4 — giving knowledge and information to the patient

- Patient and family should be informed that there is usually a continuum along cognitive senescence, subjective cognitive decline, MCI and dementia. It is crucial to define dementia and then distinguish it from the effects of senescence on memory and other cognitive functions.^21^ ^,^ ^23^
- Before communicating the diagnosis, the physician briefly discusses laboratory findings, excluding reversible causes of cognitive deficits.^21^
- The patient is told the diagnosis. It is appropriate to stress some positive aspects of AD and other dementias, such as the slow progressive course, pharmacological and non-pharmacological treatments, trials on AD and the encouragement to maintain the patient's autonomy.^8^ ^,^ ^20^ ^,^ ^21^ Try to outline the short-term changes in the patients and caregivers. Do tell them that it is difficult to predict the course of dementia.^21^
- Avoid terms such as “senile dementia”, “senility”, “forgetfulness” or “brain failure”.
- Avoid drop-by-drop information. This kind of disclosure may become more puzzling to the person with dementia^(^ ^21^ ^)^.

Step 5 — addressing the patient's emotions with empathic responses

- SPIKES’ 5th step describes the empathic responses that are also adequate for a dementia patient.^8^ ^,^ ^21^
- It is also essential to provide emotional support for the caregiver, whose distress may be even greater compared to the patient.^20^ ^,^ ^21^

Step 6 — strategy and summary

- Besides discussing the pharmacological options, the physician should comment on the available cognitive and functional rehabilitation, as well as living centers for leisure and socialization.
- It is important to mention that legal and safety issues such as lasting power of attorney, advance directives will and driving capacity need to be addressed in a future meeting, preferably in a multidisciplinary team.
- It is recommended to offer educational brochures about AD, as well as informing about caregiver's support groups, either online or community-based (e.g., Alzheimer's Association, Abraz).^20^ ^,^ ^22^ ^,^ ^23^

## DISCUSSION

SPIKE's first step is related to the setting up of the encounter. The clinician and other members of the team must prepare themselves in advance and be aware of every detail they are about to share, such as neuropsychological assessment, neuroimaging and laboratory results. Although the presence of a family member is mentioned in the SPIKES protocol under the patient's choice, many papers advise that this member or trusted person should participate in the delivery of the diagnosis of dementia.^8^ ^,^ ^21^ ^,^ ^22^ ^,^ ^25^ Not only the cognitive deficit may limit the true understanding of the disease but also it is the family who will probably be involved in the care of the patient as long as the disease advances.^21^

As dementia progresses, cognitive functions such as episodic memory, reasoning, judgement, abstraction, insight, planning and complex task management worsen. This impairment will directly affect patients’ understanding about the condition and its future implications, as well as their decision-making capacity and competency.^7^ Hence, it is vital to judge the patient's level of cognitive impairment and adapt the communication while breaking the bad news. Ideally, we should communicate the diagnosis of AD or other dementia to patients in early stages of cognitive decline, as in MCI or early dementia. These patients can still engage in an active life, making decisions about the future. In later stages of disease, the truth is not meaningful to the patient, so it will be neither of benefit nor harm. In the severely affected, disclosure is merely futile.^7^ ^,^ ^20^ ^,^ ^22^

Another important suggestion is to directly address the patient in the communication process, as a common mistake is to neglect the person with dementia and talk straightly to the caregiver.^21^ On the other hand, when more advanced clinical stages are addressed, the clinician may communicate the diagnosis to a family member or caregiver, once the patient's comprehension is limited. Like in the original SPIKES protocol, in the second step of this adapted protocol, we question the patient about his/her understanding about the memory complaints. For example, “In your point of view, what is happening with your memory? Dou you think your memory complaints may be due to a disease?”.

The third step of SPIKES is similar in the two protocols. Here, we ask the patient whether he/she would like to know the diagnosis, because the patient's right to know or not to know is a well-established priority.^7^ ^,^ ^20^ Actually, the concern about the patient's consent should begin prior to the assessment, where the possible findings for the cognitive complaints should be discussed with the patient.

It is in the fourth step that the information related to the diagnosis is provided. Differently from well-known diseases such as cancer, heart disease or diabetes, dementia syndromes such as Lewy body dementia, vascular dementia or frontotemporal dementia may sound abstract to most of the patients, yet AD is often a well-known disease to the general public. It is helpful to frame memory loss as a spectrum, with “normal aging” at one end, “dementia” at the other end, and “mild cognitive impairment” in between. We should make sure that the patient and family comprehend that dementia represents the worsening of the cognitive functions that interferes with daily life functions. Described in this way, it is easier to understand dementia as a part of a continuum of memory loss.^23^

Before sharing the diagnosis, the physician should briefly discuss laboratory findings, excluding reversible causes of cognitive deficits. The diagnosis of dementia should then be informed in short and clear terms, such as “Alzheimer's disease” and ‘dementia’. Pitfalls to be avoided in this phase include the use of jargon, meaning medical specialist terms,^21^ as well as indirect terms such as “senile dementia”, “senility”, “forgetfulness” or “brain failure”.

It is not uncommon that patients and family members have had negative experiences with others who suffered from dementia. It is appropriate to stress positive aspects of most dementias, such as the slow progressive course, treatment options that might delay decline for a while and the ongoing progress being made in dementia research.^8^ ^,^ ^20^ ^,^ ^21^ ^,^ ^23^ In the early stages of cognitive impairment, it is helpful to provide realistic hope emphasizing that much of their brain still functions well and that the patient's autonomy and quality of life will be encouraged.^20^ ^,^ ^23^

SPIKES’ 5th step describes the empathetic responses that are also adequate for the dementia scenario. Creating time for emotions has the purpose of overcoming the patient's and family's first shock and opening themselves to further explanations. Possible emotions are sadness, tearfulness, disbelief, denial, silence, anger or shock. Most people with dementia, like the majority of us, are able to control their emotions at the point of receiving news of their diagnosis. Tolerance of silence is crucial, as people with dementia may take a bit more of time to understand their feelings. The physician can help patients to identify their feelings and to reflect on them.^21^ ^,^ ^26^ It is also essential to provide emotional support for the caregiver, whose distress may be even greater than the patient's.^20^ ^,^ ^21^ ^,^ ^26^

In the final stage of this adapted protocol (Summary and Strategy), it is important to answer questions that can be raised after the disclosure. Probably, patients and caregivers do not fully retain the information given, so it is suitable to clarify it, linking the cognitive complaints and personality changes with basic information about the disease. Questions about the direct consequences of dementia may be posed, such as behavioral changes, legal issues and prognosis about functioning and independence. Once questions have been answered, it is time for discussion about follow up. Besides pharmacological options, the team should comment on the available cognitive and functional rehabilitation, as well as living centers for leisure and socialization. Recommendations about lifestyle changes are also welcome at this time.

It is recommended to offer caregiver education and support, sharing digital or written information, such as brochures, leaflets or reading suggestions, as well as informing about support groups, either online or community-based (e.g.. Abraz, Alzheimer's Association, Alzheimer's Society, Alzheimer Portugal, Corporación Alzheimer Chile, etc.).^21^ ^–^ ^23^

The definite advantage of the SPIKES protocol is its didactic approach, which has contributed to its adoption worldwide in situations of breaking bad news. It has also been taught in a large number of medical schools across Brazil, despite an unclear figure. However, some limitations to this adapted protocol may be mentioned. The SPIKES model relates to a singular event of disclosing bad news whereas for individuals with dementia, additional time may be necessary to make sense of the information.^31^ The initial meeting is often overwhelming; thus, it should be a good practice to arrange follow-up sessions with other members of the multi-disciplinary team, in which questions such as “Perception of receiving the diagnosis of dementia”, “Diagnostic expectations: confirmation or surprise?” “Did the person with dementia and caregiver share the diagnosis with others?’ would be discussed.^21^ ^,^ ^23^

Another issue is that the SPIKES framework as well as other existing protocols on breaking bad news may not fully support the disclosure of a dementia diagnosis in the same way they do not work for persons with intellectual disabilities. In people with learning disabilities and those living with dementia, there are many pieces of complex information to make sense of.^31^ Someone's framework of knowledge grows over time. However, when people have dementia, their framework is shrinking, and their chunks of knowledge are gradually shifting. The boundaries between background knowledge and “what is happening right now” may become blurred.^32^ In a way to address the disclosure of dementia in people with learning disabilities, a new model targeting the process of building a foundation of knowledge was published in 2013.^32^ This tool is a stepped approach that includes building up pieces of complex information over time, understanding how a person gains understanding of an area that is difficult to operationalize, engaging people from the various systems surrounding the person involved in the diagnostic process and finally giving support to the patient, family and caregivers considering the impact of the diagnosis on their life.^31^ ^,^ ^32^ Although this model helps people with intellectual disabilities on the transitional adjustment over a period of time after the diagnosis, it is a time-consuming and quite redundant tool to be used in people with MCI or early dementia with previously normal cognition.

Cross-cultural differences between Latin America and countries in North America and Europe must also be addressed. In the former, it is common to deal with relatives or caregivers who ask that the diagnosis be withheld from the patient, under the unrealistic thoughts to protect the person with dementia from fear and distress, or even a catastrophic reaction. In the SPIKES framework, there is no place for addressing this issue with the family, so maybe we should further discuss the importance of incorporating a pre-disclosure meeting with these members to discuss their thoughts and the benefits of truth-telling to patients in early stages of cognitive dysfunction.

Diagnostic disclosure of dementia is particularly challenging. However, it is considered a fundamental step in the care of patients and their relatives and caregivers. The lack of specific guidelines may contribute to the low rates or inappropriate communication processes when delivering a diagnosis of dementia is considered. The SPIKES protocol is a widely used tool to break bad news, although it was designed for cognitively intact people. The suggested adaptations in the SPIKES-D protocol may facilitate the delivery of the diagnosis to persons with AD and other dementias, encompassing current guidelines about this theme. To widen its applicability in dementia care settings, it is crucial to implement the teaching of communication skills in undergraduate medical schools, along with SPIKES-D training in residence programs such as geriatrics, neurology and psychiatry. This tool must still be analyzed by a jury of specialists and validated after being tested in a sample population. Possible outcomes to be investigated include patients’ and caregivers’ satisfaction with the communication process, psychological complaints arising after diagnosis disclosure and whether delivering a dementia diagnosis through the SPIKES-D protocol results in better compliance to treatment.

## Appendix 1 SPIKES-D protocol.

**Table:**

SPIKES-D PROTOCOL A PROPOSAL TO ADAPT THE SPIKES PROTOCOL TO DELIVER THE DIAGNOSIS OF DEMENTIA
STEP 1: **SETTING UP** THE INTERVIEW Prepare yourself for the task; Manage time and arrange for some privacy; Sit down and make connection with the patient (make use of non-verbal communication); Have a trusted family member on the disclosure meeting; Avoid social talk or long introductions; Explain the reason of the meeting; Deliver the diagnosis preferably in a multidisciplinary approach.
STEP 2: ASSESSING THE PATIENTS’ **PERCEPTION** Identify the extent of cognitive impairment and degree of insight of the patient. An effective communication still occurs in the early stages of dementia or MCI (Mild Cognitive Impairment); Directly address the person with dementia in the disclosure meeting; Comprehend hopes and expectations; Identify what the patient already knows or suspects about the medical condition; Examples: “Do you know what is causing your memory problems”? “Do you think they may be due to a disease”?
STEP 3: OBTAINING THE PATIENTS’ **INVITATION** Identify whether the patient desires full information about diagnosis and prognosis; If the patient declines to be told the diagnosis, the clinician should arrange a future talk with a family member; An appropriate way to invite him should be: “Mr. ___, we have analysed the cognitive tests and the laboratory results you have been through. It seems we already have an idea of what is happening to your memory. Would you like to know it”?
STEP 4: GIVING **KNOWLEDGE** AND INFORMATION TO THE PATIENT Use adequate language concerning the patient's educational and cultural background; Avoid technical words (jargons); Give information in small chunks and check periodically as to the patient's understanding; Tell the truth about the condition. Avoid discouraging phrases such as “there is nothing we can do for you”; Explain the continuum along cognitive senescence, subjective cognitive decline, MCI and dementia. Define what dementia is; Discuss laboratory findings, excluding reversible causes of cognitive decline; Disclose the diagnosis. Avoid terms such as “senile dementia”, “senility”, “forgetfulness” or “brain failure”; Try to stress some positive aspects of AD and some dementias, such as the slow progressive course, pharmacological and non-pharmacological treatments, trials on AD and the encouragement to maintain the patient's autonomy; Try to outline the short-term changes in the patients and caregivers. Tell them that it is difficult to predict the course of dementia; Avoid drop-by-drop information. This kind of disclosure may become more puzzling to the person with dementia.
STEP 5: ADDRESSING THE PATIENT’S **EMOTIONS** WITH EMPATHIC RESPONSES Observe for any emotion on the part of the patient (sadness, silence, anger, shock, denial); Give him time to express his feelings. Respect the silence; Demonstrate empathy; Help the patient to understand his/her emotions; Provide emotional support to the caregiver.
ETAPA 6: STRATEGY AND SUMMARY Clarify any questions; Share responsibility with the patient for decision-making; Discuss a treatment plan; Besides offering pharmacological treatment, the physician should comment on cognitive and functional rehabilitation, as well as living centres for leisure and socialization; Mention legal and safety issues which need to be addressed in a future meeting, preferably in a multidisciplinary team; Offer educational brochures about AD, as well as caregiver's support groups, (e.g., Alzheimer's Association, Alzheimer's UK, Abraz); Ensure long-term follow-up;

* The original SPIKES protocol is written in regular letters and the suggested adaptations to SPIKES-D in **bold**.^26^

## References

[1] United Nations World Population Prospects: The 2017 Revision: Key Findings and Advance Tables.

[2] Winblad B, Amouyel P, Andrieu S, Ballard C, Brayne C, Brodaty H (2016). Defeating Alzheimer's disease and other dementias: a priority for European science and society. Lancet Neurol.

[3] Custodio N, Wheelock A, Thumala D, Slachevsky A (2017). Dementia in Latin America: epidemiological evidence and implications for public policy. Front Front Aging Neurosci.

[4] Bamford C, Lamont S, Eccles M, Robinson L, May C, Bond J (2004). Disclosing a diagnosis of dementia: a systematic review. Int J Geriatr Psychiatry.

[5] Werner P, Karnieli-Miller O, Eidelman S (2013). Current knowledge and future directions about the disclosure of dementia: a systematic review of the first decade of the 21st century. Alzheimers Dement.

[6] Raicher I, Shimizu MM, Takahashi DY, Nitrini R, Caramelli P (2008). Alzheimer's disease diagnosis disclosure in Brazil: a survey of specialized physicians’ current practice and attitudes. Int Psychogeriatr.

[7] Pinner G, Bouman WP (2002). To tell or not to tell: on disclosing the diagnosis of dementia. Int Psychogeriatr.

[8] Selmès J, Derouesné C (2004). Réflexions sur l'annonce du diagnostic de la maladie d'Alzheimer. Psychol Neuropsychiatr Vieil.

[9] Philips J, Pond CD, Paterson NE, Howell C, Shell A, Stocks NP (2012). Difficulties in disclosing diagnosis dementia: a qualitative study in general practice. Br J Gen Pract.

[10] Vivela LP, Caramelli P (2006). A Alzheimer's disease as viewed by relatives of patients at public and private clinics. Rev Assoc Med Bras (1992).

[11] Hellström I, Torres S (2013). A wish to know but not always tell–couples living with dementia talk about disclosure preferences. Aging Ment Health.

[12] Dautzenberg PL, van Marum RJ, van der Hammen R, Paling HA (2003). Patients and families desire a patient to be told the diagnosis of dementia: a survey by questionnaire on a Dutch memory clinic. Int J Geriatr Psychiatry.

[13] Pinner G, Bouman WP (2003). Attitudes of patients with mild dementia and their carers towards disclosure of the diagnosis. Int Psychogeriatr.

[14] Shimizu MM, Raicher I, Takahashi DY, Caramelli P, Nitrini R (2008). Disclosure of the diagnosis of Alzheimer disease. Caregivers’ opinions in a Brazilian Sample. Arq Neuro-Psiquiatr.

[15] Tracy CS, Drummond N, Ferris LE, Globerman J, Hébert PC, Pringle DM (2004). To tell or not to tell? Professional and lay perspectives on the disclosure of personal health information in community-based dementia care. Can J Aging.

[16] Karnieli-Miller O, Werner P, Aharon-Peretz J, Eidelman S (2007). Dilemmas in the (un) veiling of the diagnosis of Alzheimer's disease: walking an ethical and professional tight rope. Patient Educ Couns.

[17] Carpenter B, Dave J (2004). Disclosing a dementia diagnosis: a review of opinion and practice, and a proposed research agenda. Gerontologist.

[18] Rice K, Warner N (1994). Breaking the bad news: what do psychiatrists tell patients with dementia about their illness?. Int J Geriatr Psychiatry.

[19] van Hout HP, Vernooij-Dassen MJ, Jansen DA, Stalman WA (2006). Do general practitioners disclose correct information to their patients suspected of dementia and their caregivers? A prospective observational study. Aging Ment Health.

[20] Fuentes PR, Prato JA (2012). Comunicación diagnóstica en enfermedad de Alzheimer: reflexión y propuesta. Rev Méd Chile.

[21] Dersksen E, Vernooij-Dassen M, Scheltens P, Olde-Rikkert M (2006). A model for disclosure diagnosis dementia. Dementia.

[22] Pinner G, Bouman WP (2003). What should we tell people about dementia?. Adv Psychiatr Treat.

[23] Lee L, Weston WW (2011). Disclosing a diagnosis of dementia. Helping learners to break bad news. Can Fam Physician.

[24] Lecouturier J, Bamford C, Hughes JC, Francis JJ, Foy R, Johnston M, Eccles MP (2008). Appropriate disclosure of a diagnosis of dementia: identifying the key behaviours of ‘best practice’. BMC Health Serv Res.

[25] Bennett CE, De Boos D, Moghaddam NG (2019). Developing a tool to support diagnostic delivery of dementia. Dementia (London).

[26] Baile WF, Buckman R, Lenzi R, Glober G, Beale EA, Kudelka AP (2000). SPIKES - A six-step protocol for delivering bad news: application to the patient with cancer. Oncologist.

[27] VandeKieft GK (2001). Breaking bad news. Am Fam Physician.

[28] Park I, Gupta A, Mandani K, Haubner L, Peckler B (2010). Breaking bad news education for emergency medicine residents: a novel training module using simulation with the SPIKES protocol. J Emerg Trauma Shock.

[29] Setubal MSV, Goncalves AV, Rocha SR, Amaral EM (2017). Breaking bad news training program based on video reviews and SPIKES strategy: what do perinatology residents think about it?. Rev Bras Ginecol Obstet.

[30] Rosenbaum ME, Ferguson KJ, Lobas JG (2004). Teaching medical students and residents skills for delivering bad news: review of strategies. Acad Med.

[31] Murphy G, Gair E (2013). Communicating a diagnosis of dementia. FPOP Newsletter.

[32] Tuffrey-Wijne I, Watchman K (2015). Breaking bad news to people with learning disabilities and dementia. Learn Disabil Pract.

